# Incremental burden of comorbid major depressive disorder in patients with type 2 diabetes or cardiovascular disease: a retrospective claims analysis

**DOI:** 10.1186/s12913-021-06802-9

**Published:** 2021-08-06

**Authors:** Anne Kangethe, Debra F. Lawrence, Maëlys Touya, Lambros Chrones, Michael Polson, Themmi Evangelatos

**Affiliations:** 1 Magellan Method, a Division of Magellan Rx Management, 35 Landsdowne St, Scottsdale, AZ 85251 USA; 2 Takeda Pharmaceuticals U.S.A., Inc., 95 Hayden Ave, Lexington, MA 02421 USA; 3 Lundbeck LLC, 6 Parkway North, Deerfield, IL 60015 USA

**Keywords:** Depression, Comorbidities, Health economics, Healthcare utilization, Pharmacoeconomics

## Abstract

**Background:**

The estimated prevalence of comorbid major depressive disorder (.

MDD) is 11% in patients with type 2 diabetes (T2D) and 15–20% in those with cardiovascular disease (CVD). Comorbid MDD continues to be a significant source of economic burden to the healthcare system.

**Methods:**

We assessed the incremental healthcare burden of comorbid MDD in patients with T2D or CVD. This real-world, retrospective, administrative claims study analyzed commercially insured adults with T2D or CVD diagnosed on at least 2 separate claims within 12 months of each other (between January 1, 2011, and September 30, 2018). CVD included congestive heart failure, peripheral vascular disease, coronary heart disease, and cerebrovascular disease. The study compared patients with and without MDD with either T2D or CVD. Study assessments included all-cause healthcare resource utilization (proportion of patients with hospitalization, emergency department [ED] visits, and outpatient visits) and cost.

**Results:**

Patients were matched by propensity score for demographics and baseline characteristics, resulting in similar baseline characteristics for the respective subcohorts. After matching, 22,892 patients with T2D (11,446 each with and without MDD) and 28,298 patients with CVD (14,149 each with and without MDD) were included.

At follow-up, patients with T2D and MDD had significantly higher rates of hospitalization (26.1% vs 17.4%, *P* < 0.0001) and ED visits (55.3% vs 43.0%, *P* < 0.0001) than those observed in patients without MDD. The total cost for patients with T2D and MDD at follow-up was significantly higher than for those without MDD ($16,511 vs $11,550, *P* < 0.0001). Similarly, at follow-up, patients with CVD and MDD had significantly higher rates of hospitalization (45.4% vs 34.1%, *P* < 0.0001) and ED visits (66.5% vs 55.4%, *P* < 0.0001) than those observed in patients without MDD. Total cost at follow-up for patients with CVD and MDD was significantly higher than for those without MDD ($25,546 vs $18,041, *P* < 0.0001).

**Conclusions:**

Patients with either T2D or CVD and comorbid MDD have higher total all-cause healthcare utilization and cost than similar patients without MDD. Study findings reinforce the need for appropriate management of MDD in patients with these comorbid diseases, which in turn may result in cost reductions for payers.

**Trial registration:**

Not applicable.

**Supplementary Information:**

The online version contains supplementary material available at 10.1186/s12913-021-06802-9.

## Background

Major depressive disorder (MDD) is a common condition that is estimated to affect more than 17 million adults in the United States (US) [1]. Individuals with chronic medical conditions have an even higher prevalence of MDD than the average population [2]. This trend is also observed in patients with cardiovascular disease (CVD) who have been shown to have an MDD prevalence of 2 to 3 times higher than that of the general population [3]. Possible mechanisms that may provide a plausible link between CVD and MDD include inflammation, endothelial dysfunction, increased platelet activity and aggregation, neurohormonal and autonomic nervous system dysfunction, effects of brain-derived neurotrophic factor and related factors, and behavioral factors [4].

Similarly, MDD is a major public health issue among patients with type 2 diabetes (T2D). The prevalence of comorbid T2D is greater in patients with MDD than in the general population [5]. There is also increasing evidence that depression and diabetes may have common pathways, such as cytokine-mediated inflammatory responses and dysregulation of the hypothalamic-pituitary-adrenal (HPA) axis [6].

On the basis of the National Health and Nutrition Examination Survey data from 2013 to 2016, an estimated 10% of adults in the US (26 million) have a diagnosis of diabetes and 48% of adults aged ≥20 years (121.5 million in 2016) have CVD [7]. The estimated prevalence of comorbid MDD is 11% in patients with diabetes and 15 to 20% in patients with CVD [4, 8, 9]. In addition, 31 to 45% of patients with coronary artery disease, unstable angina, or myocardial infarction suffer from clinically significant depressive symptoms [4].

Comorbid MDD continues to be a significant source of economic burden to the healthcare system, with costs for adults estimated at $210.5 billion in 2010 [10]. In the limited studies that have been conducted, patients with T2D or CVD who also have depression have been shown to have higher total costs than costs for similar patients without depression. In fact, total costs for patients with diabetes and depression are 2 to 4½ times the costs for patients without depression, and these costs increase as depressive symptoms become more severe [11]. Similarly, among women with suspected myocardial infarction, patients with depression have been associated with a 15 to 53% increase in 5-year cardiovascular-related costs compared with those without depression [12].

As further evidence of increased utilization and costs, specifically for patients with comorbid MDD, a review of the literature revealed a prior published study that assessed use of healthcare resources among individuals with T2D with and without comorbid MDD [13]. This study found that between 2005 and 2006, patients with T2D and comorbid MDD utilized significantly more healthcare services, such as hospitalizations, emergency department (ED) visits, and outpatient visits (*P* < 0.0001) than the services used by patients with T2D without comorbid MDD. Moreover, mean total costs per year were $8470 higher for patients with comorbid MDD than for those without comorbid MDD ($19,707 vs $11,237, *P* < 0.0001) [13].

The substantial burden of comorbid MDD among patients with T2D or CVD is of significant concern, and additional data are necessary to assess its impact in real-world populations. Therefore, the objective of this hypothesis-testing study was to assess the incremental healthcare burden of MDD in patients with T2D or CVD in the US.

## Methods

### Study design

This was a real-world, retrospective cohort study that analyzed patients enrolled in commercial health plans using administrative medical and pharmacy claims data. The study period was defined as claims that were incurred between January 1, 2011, and September 30, 2018. The study utilized data from the medical/pharmacy database of Magellan Rx Management, which consists of claims data submitted by health plans with contracts to receive various services from Magellan (Additional file 1: Fig. S1). This database represents populations in regional health plans and contains adjudicated paid claims. The data that were analyzed represent submissions by the providers and are validated within tolerance limits. These data included information commonly required in institutional, professional, and pharmacy claims, such as dates of service, provider information, procedure codes, drug prescriptions, and financial information. A quality check was performed by the authors involved in analysis of the data for each individual health plan, prior to their inclusion in the study. In addition, consistency among monthly claim counts, patient counts, and allowed and paid amount totals were verified.

This study is exempt from Institutional Review Board requirements because it includes analyses of administrative claims datasets and fulfills the following exemption requirements: (1) the study does not include any individually identifiable health information, (2) the authors are authorized by the medical/pharmacy database manager of Magellan Rx Management to use the Protected Health Information (PHI) in conducting analyses performed under the “Treatment, Payment, Health Care Operations” provision of the Health Insurance Portability and Accountability Act of 1996 (HIPAA), and (3) the authors are bound by Magellan’s confidentiality standards that protect individually identifiable health information, including HIPAA-compliant procedures for storage, transmission, release, and disposal of PHI.

### Study population

For inclusion in the study, patients were required to have a diagnosis of T2D or CVD on at least 2 separate claims within 12 months of each other; CVD included congestive heart failure, peripheral vascular disease, coronary heart disease, and cerebrovascular disease.

Patients were also required to be ≥18 years of age on the index date, which was defined as the first medical claim with a T2D or CVD diagnosis code during the identification period. Continuous enrollment in both medical and pharmacy benefits for at least 12 months before the index date through 12 or more months after the index date was also a requirement. Patients were excluded from the study if they had a diagnosis for schizophrenia or bipolar disorder at any time before the end of their healthcare plan enrollment or end of data availability, or if they became pregnant at any time during the study period.

Those patients who met the inclusion criteria were divided into 4 subcohorts, which were defined as follows: (1) patients with T2D and comorbid MDD; (2) patients with T2D without comorbid MDD; (3) patients with CVD and comorbid MDD; and (4) patients with CVD without comorbid MDD. For the 2 MDD subcohorts, patients must have had at least 2 separate diagnoses of MDD (based on the *International Classification of Diseases, Ninth or Tenth Revision, Clinical Modification [ICD-9/10-CM]*), with 1 of the 2 diagnoses occurring in the period between 1 year before and 1 year after the index date. Patients were included in the MDD cohort if their claims included the *ICD-9-CM* codes 296.2x, 296.3x, 300.4, or 311, or the *ICD-10-CM* codes F32.xx, F33.xx, and F34.1.

### Endpoints and statistical analyses

Patients with comorbid MDD were matched to patients without comorbid MDD by using propensity scores; the propensity scores model used matching variables that included age group, sex, region, plan type, and select comorbidities. The comorbidities were chosen based on a literature review and clinical advice, and included the following: substance/alcohol abuse disorder; T2D; hyperlipidemia; obesity; hypertension; chronic kidney disease; chronic heart disease; congestive heart failure; cerebrovascular disease; peripheral vascular disease; cancer; anxiety disorders; sleeping disorders; chronic obstructive pulmonary disease; Parkinson’s disease; multiple sclerosis; and gastrointestinal disorders.

This study assessed healthcare resource utilization and costs among patients with comorbid MDD compared with the matched patients without comorbid MDD. Statistical differences between groups in healthcare resource utilization and costs were determined using t tests. The following endpoints were analyzed: (1) annual all-cause healthcare resource utilization and costs during the baseline period (defined as the 12-month period prior to the index date) and (2) annual all-cause healthcare resource utilization and costs during follow-up (the period of 12 or more months after the index date), stratified by service type (including the proportions of patients requiring hospitalization; emergency department ED, laboratory, or outpatient visits; and medical and pharmacy costs). All costs were adjusted to 2018 dollars using the change in the medical care component of the Consumer Price Index [14].

All analyses were performed using SAS software version 9.4 (SAS Institute, Cary, NC).

## Results

### Patient disposition and demographics

Medical and pharmacy claims data were available during the study period from a total of 12.8 million patients. Of these, 969,944 (7.6%) had a diagnosis of T2D on at least 2 separate claims within 12 months of each other, and 890,819 (7.0%) had a diagnosis of CVD on at least 2 separate claims within 12 months of each other. Applying the remaining eligibility criteria (age, continuous enrollment, and exclusion criteria) reduced the population to 146,282 patients with T2D (11,753 with comorbid MDD and 134,529 without comorbid MDD) and 136,632 patients with CVD (14,750 with comorbid MDD and 121,882 without comorbid MDD) (Additional file 2: Table S1). Prior to matching, significant differences in demographic characteristics were evident between patients with and without comorbid MDD for both the T2D and CVD populations.

Following matching, the respective subcohorts with and without comorbid MDD had similar demographics **(**Table 1**)**. Matching the patients with comorbid MDD to patients without comorbid MDD using propensity scores resulted in a final study population of 22,892 patients with T2D (11,446 with comorbid MDD and 11,446 without comorbid MDD) and 28,298 patients with CVD (14,149 with comorbid MDD and 14,149 without comorbid MDD).

**Table 1:** Patient demographics after matching

Characteristic	Patients with T2D				Patients with CVD			
	All patients (***n*** = 22,892)	With comorbid MDD (***n*** = 11,446)	Without comorbid MDD (***n =*** 11,446)	***P*** value	All patients (***n =*** 28,298)	With comorbid MDD (***n*** = 14,149)	Without comorbid MDD (***n =*** 14,149)	***P*** value
**Age, years**								
Mean (SD)	52.8 (9.8)	51.3 (9.4)	51.2 (9.5)	0.08	52.8 (9.3)	52.8 (9.4)	52.9 (9.3)	0.59
**Age group, years, n (%)**								
18–29	580 (2.5)	301 (2.6)	279 (2.4)	0.45	667 (2.4)	345 (2.4)	322 (2.3)	0.83
30–39	1820 (8.0)	939 (8.2)	881 (7.7)		1630 (5.8)	810 (5.7)	820 (5.8)	
40–49	6030 (26.3)	3004 (26.2)	3026 (26.4)		5872 (20.8)	2928 (20.7)	2944 (20.8)	
50–59	10,015 (43.7)	4970 (43.4)	5045 (44.1)		13,303 (47.0)	6627 (46.8)	6676 (47.2)	
60–69	4266 (18.6)	2133 (18.6)	2133 (18.6)		6408 (22.6)	3217 (22.7)	3191 (22.6)	
70–79	174 (0.8)	94 (0.8)	80 (0.7)		375 (1.3)	199 (1.4)	176 (1.2)	
≥80	7 (0.0)	5 (0.0)	2 (0.0)		43 (0.2)	23 (0.2)	20 (0.1)	
**Sex, n (%)**								
Female	13,947 (60.9)	6969 (60.9)	6978 (61.0)	0.91	16,809 (59.4)	8379 (59.2)	8430 (59.6)	0.55
Male	8945 (39.1)	4477 (39.1)	4468 (39.0)		11,489 (40.6)	5770 (40.8)	5719 (40.4)	
**Plan type, n (%)**								
PPO/POS	19,337 (84.5)	9621 (84.1)	9716 (84.9)	0.19	23,528 (83.1)	11,724 (82.9)	11,804 (83.4)	0.44
HMO	1626 (7.1)	843 (7.4)	783 (6.8)		2504 (8.8)	1271 (9.0)	1233 (8.7)	
Other	1929 (8.4)	982 (8.6)	947 (8.3)		2266 (8.0)	1154 (8.2)	1112 (7.9)	

Statistical differences were assessed using *t* tests

*CVD* cardiovascular disease, *HMO* health maintenance organization, *MDD* major depressive disorder, *POS* point of service, *PPO* preferred provider organization, *SD* standard deviation, *T2D* type 2 diabetes

### Healthcare resource utilization and costs during the baseline period

The results presented here are for matched patients. During the baseline period, significantly more patients with T2D or CVD and comorbid MDD required hospitalization, ED visits, or outpatient visits than did patients without comorbid MDD (all *P* < 0.0001; Table 2).

**Table 2:** Healthcare resource utilization and annual costs during the baseline period

	Patients with T2D				Patients with CVD		
		With comorbid MDD (***n =*** 11,446)	Without comorbid MDD (***n =*** 11,446)	***P*** value	With comorbid MDD (***n =*** 14,149)	Without comorbid MDD (***n =*** 14,149)	***P*** value
**Baseline average duration of analysis, months**							
Mean (SD)		43.1 (28.9)	43.1 (28.0)	0.99	42.8 (29.8)	43.4 (29.6)	0.11
**All-cause healthcare resource utilization during the baseline period, n (%)**							
Proportion of patients requiring:							
Hospitalizations		1222 (10.7)	872 (7.6)	***< 0.0001***	2250 (15.9)	1606 (11.4)	***< 0.0001***
ED visits		4368 (38.2)	3890 (34.0)	***< 0.0001***	7121 (50.3)	6431 (45.5)	***< 0.0001***
Outpatient visits		10,777 (94.2)	10,606 (92.7)	***< 0.0001***	13,887 (98.1)	13,786 (97.4)	***< 0.0001***
**Annual all-cause costs during the baseline period, $**							
Pharmacy							
Mean (SD)		1859 (6203)	1206 (4265)	***< 0.0001***	2498 (6931)	1775 (7679)	***< 0.0001***
Medical							
Mean (SD)		6127 (20,069)	4484 (15,874)	***< 0.0001***	8112 (22,857)	6335 (23,142)	***< 0.0001***
Total							
Mean (SD)		7986 (22,158)	5689 (17,467)	***< 0.0001***	10,610 (25,425)	8110 (25,077)	***< 0.0001***

Statistical differences were assessed using *t* tests. Significant *P* values (< 0.05) are shown in bold italic.

*CVD* cardiovascular disease, *ED* emergency department, *MDD* major depressive disorder, *SD* standard deviation, *T2D* type 2 diabetes

During the baseline period, mean annual total costs (inclusive of pharmacy and medical costs) were all significantly greater for patients with T2D or CVD and comorbid MDD than for matched patients without comorbid MDD (all *P* < 0.0001; Table 2**).**

### Healthcare resource utilization and costs during follow-up

During the follow-up period, significantly more patients with T2D and comorbid MDD required hospitalization, ED visits, or outpatient visits than did patients without comorbid MDD (all *P* ≤ 0.0025; Table 3). Similarly, significantly more patients with CVD and comorbid MDD required hospitalization or ED visits than did patients without comorbid MDD (both *P* < 0.0001; Table 3). The mean numbers of hospitalizations and ED, laboratory, and outpatient visits, the mean number of prescriptions per patient, and the mean length of stay during follow-up were also significantly higher in patients with T2D or CVD and comorbid MDD than in patients without comorbid MDD (all *P* < 0.0001; Table 3).

**Table 3:** All-Cause healthcare resource utilization during the follow-up period

	Patients with T2D			Patients with CVD		
	With comorbid MDD (***n =*** 11,446)	Without comorbid MDD (***n =*** 11,446)	***P*** value	With comorbid MDD (***n =*** 14,149)	Without comorbid MDD (***n =*** 14,149)	***P*** value
**Average follow-up time, months**						
Mean (SD)	33.1 (16.7)	32.0 (16.2)	***< 0.0001***	29.7 (14.0)	29.2 (13.8)	***0.0016***
**Proportion of patients requiring, n (%):**						
Hospitalizations	2989 (26.1)	1986 (17.4)	***< 0.0001***	6419 (45.4)	4826 (34.1)	***< 0.0001***
ED visits	6328 (55.3)	4918 (43.0)	***< 0.0001***	9411 (66.5)	7832 (55.4)	***< 0.0001***
Outpatient visits	11,443 (100.0)	11,429 (99.9)	***0.0025***	14,146 (100.0)	14,144 (100.0)	0.73
**Hospitalizations per patient**						
Mean (SD)	0.3 (0.8)	0.1 (0.4)	***< 0.0001***	0.5 (1.2)	0.3 (0.8)	***< 0.0001***
**Length of stay, days**						
Mean (SD)	1.6 (7.6)	0.7 (3.3)	***< 0.0001***	3.6 (11.3)	1.8 (6.4)	***< 0.0001***
**ED visits per patient**						
Mean (SD)	0.9 (2.5)	0.6 (1.9)	***< 0.0001***	1.3 (2.8)	0.9 (2.8)	***< 0.0001***
**Laboratory visits per patient**						
Mean (SD)	3.9 (5.0)	3.2 (3.8)	***< 0.0001***	4.5 (5.6)	3.7 (4.9)	***< 0.0001***
**Outpatient visits per patient**						
Mean (SD)	21.4 (18.6)	14.4 (13.8)	***< 0.0001***	26.5 (21.3)	18.5 (16.5)	***< 0.0001***
Primary care visits						
Mean (SD)	4.9 (4.2)	4.1 (4.0)	***< 0.0001***	5.0 (4.6)	4.0 (4.1)	***< 0.0001***
Psychiatrist visits						
Mean (SD)	1.1 (3.1)	0.1 (1.1)	***< 0.0001***	1.2 (3.5)	0.1 (0.9)	***< 0.0001***
Behavioral therapy visits						
Mean (SD)	3.3 (8.9)	0.5 (3.0)	***< 0.0001***	3.6 (8.8)	0.6 (3.8)	***< 0.0001***
**Prescriptions per patient**						
Mean (SD)	6.1 (7.8)	4.4 (6.0)	***< 0.0001***	7.4 (8.6)	5.2 (6.5)	***< 0.0001***

Statistical differences were assessed using *t* tests. Significant *P* values (< 0.05) are shown in bold italic.

*CVD* cardiovascular disease, *ED* emergency department, *MDD* major depressive disorder, *SD* standard deviation, *T2D* type 2 diabetes

**Fig. 1:**
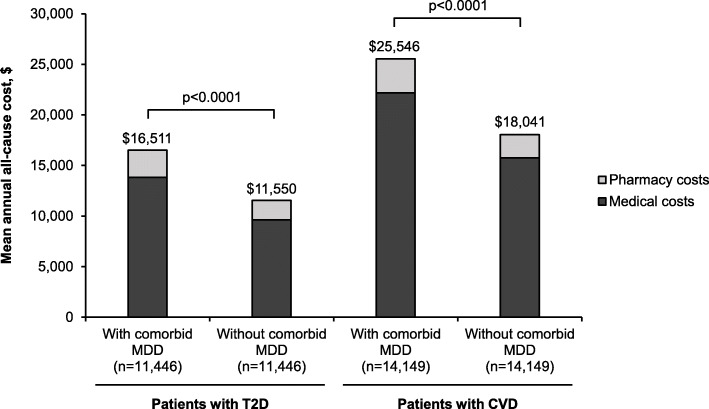
Total annual all-cause cost in patients with T2D or CVD with/without comorbid MDD during follow-up. Statistical differences were assessed using *t* tests. CVD cardiovascular disease, MDD major depressive disorder, T2D type 2 diabetes

During follow-up, mean annual all-cause costs stratified by service type were all significantly greater for patients with T2D or CVD and comorbid MDD than for patients without comorbid MDD (*P* < 0.0001 for all but “Other Costs” where *P* = 0.0015; Table 4). The mean total annual all-cause cost (pharmacy and medical) was greater by $4961 in patients with T2D and comorbid MDD than in matched patients without comorbid MDD ($16,511 vs $11,550, *P* < 0.0001; Table 4 and Fig. 1). Similarly, the mean total annual all-cause cost (pharmacy and medical) was greater by $7505 in patients with CVD and comorbid MDD than in matched patients without comorbid MDD ($25,546 vs $18,041, *P* < 0.0001; Table 4 and Fig. 1).

**Table 4:** Annual all-cause costs during the follow-up period

Annual all-cause cost, $	Patients with T2D				Patients with CVD			
	All patients (***n =*** 22,892)	With comorbid MDD (***n =*** 11,446)	Without comorbid MDD (***n =*** 11,446)	***P*** value	All patients (***n =*** 28,298)	With comorbid MDD (***n =*** 14,149)	Without comorbid MDD (***n =*** 14,149)	***P*** value
**Hospitalization costs**								
Mean (SD)	1120 (7851)	1453 (9849)	788 (5105)	***< 0.0001***	2322 (10,245)	2865 (12,120)	1778 (7903)	***< 0.0001***
**ED costs**								
Mean (SD)	878 (3387)	1087 (4321)	669 (2047)	***< 0.0001***	1440 (3711)	1746 (4508)	1134 (2652)	***< 0.0001***
**Laboratory costs**								
Mean (SD)	700 (2208)	799 (2721)	601 (1527)	***< 0.0001***	818 (2279)	930 (2723)	705 (1716)	***< 0.0001***
**Outpatient costs**								
Mean (SD)	7116 (21,936)	8283 (20,686)	5950 (23,061)	***< 0.0001***	11,005 (26,441)	12,658 (28,902)	9351 (23,611)	***< 0.0001***
**Other costs**								
Mean (SD)	1906 (14,082)	2202 (14,950)	1610 (13,150)	***0.0015***	3383 (16,220)	3989 (18,373)	2778 (13,707)	***< 0.0001***
**Medical costs**								
Mean (SD)	11,720 (31,188)	13,823 (32,023)	9618 (30,186)	***< 0.0001***	18,968 (38,326)	22,189 (42,317)	15,746 (33,561)	***< 0.0001***
**Pharmacy costs**								
Mean (SD)	2310 (7602)	2688 (8606)	1932 (6423)	***< 0.0001***	2826 (8409)	3358 (9233)	2295 (7458)	***< 0.0001***
**Total costs (pharmacy and medical)**								
Mean (SD)	14,031 (33,286)	16,511 (34,415)	11,550 (31,928)	***< 0.0001***	21,794 (40,557)	25,546 (44,796)	18,041 (35,425)	***< 0.0001***

Statistical differences were assessed using *t* tests. Significant *P* values (< 0.05) are shown in bold italic.

*CVD* cardiovascular disease, *ED* emergency department, *MDD* major depressive disorder, *SD* standard deviation, *T2D* type 2 diabetes

## Discussion

In this study, patients with either T2D or CVD and comorbid MDD had higher total all-cause healthcare utilization and costs than those reported for similar patients without comorbid MDD. Significantly more patients with T2D or CVD and comorbid MDD required hospitalization and ED visits than did matched patients without comorbid MDD. Mean annual all-cause costs stratified by service type were all significantly greater for patients with T2D or CVD and comorbid MDD than for matched patients without comorbid MDD.

Evidence from previous studies has similarly demonstrated higher healthcare utilization and costs among patients with either T2D or CVD and comorbid depression than in those without depression. As noted, earlier studies found that costs for patients with diabetes and depression were about twice those for patients without depression [11, 13]; in women with suspected myocardial infarction, cardiovascular-related costs were higher for patients with depression than for those without depression [12]. Our findings are consistent with the existing literature and add data from a large population of patients with T2D or CVD and comorbid MDD, further adding to the published base of evidence.

One possible explanation for our findings is that patients with poorly managed MDD are less able to effectively manage their comorbid chronic conditions. Consistent with this speculation, comorbid MDD and diabetes have been associated with suboptimal glycemic control and an increase in diabetes-related complications, which may be due to the fact that MDD can impair a patient’s self-management of T2D [15]. If this is true, more effective treatment of depressive symptoms may enable patients to better manage their chronic diseases. Consistent with this idea, a recently published study found that among 1568 patients with comorbid MDD and CVD (prior myocardial infarction or stroke), those who received adequate treatment for MDD had significantly fewer all-cause hospitalizations, outpatient visits, CVD-related ED visits, and CVD-related outpatient visits than those who received insufficient treatment for their MDD [16]. In this same study, patients with adequate MDD treatment also incurred significantly lower mean all-cause outpatient costs ($2055 vs $2820 per-patient per-year; *P* < 0.001), CVD-related hospitalization costs ($17,756 vs $21,485 per-patient per-year; *P* = 0.04), and CVD-related outpatient costs ($434 vs $520 per-patient per-year; *P* = 0.03) [16].

Thus, clinicians should be especially conscious of the importance of effective management of MDD in patients who also have comorbid physical disorders, both to improve patients’ mental health as well as their ability to manage their comorbid conditions. Key to effective management of any chronic condition is patients’ adherence to prescribed therapy. Comorbid MDD has been previously shown to have a negative impact on treatment adherence for patients with CVD or T2D, which has been associated with poorer outcomes in some studies [15, 17]. Medication nonadherence in patients with comorbid MDD and CVD occurs at a rate more than double that of patients without MDD [17].

Adherence to antidepressant therapy in turn supports adherence to therapy for comorbid physical conditions and better physical health outcomes. For example, among patients with comorbid T2D and MDD, those with better adherence and persistence to antidepressants have demonstrated improved adherence to oral antidiabetic medications, better glycemic control, and lower total all-cause and medical costs [15]. In addition to the impact of adherence to antidepressant therapy in assisting patients to better manage comorbid health conditions, depression and both T2D and CVD also share certain common physiological pathways [4, 6]. Thus, effective management of MDD may also have direct benefits on the physical health disorder. In summary, effective management of MDD has the potential to improve physical health outcomes and reduce healthcare utilization and costs among patients with comorbid diseases.

### Limitations

Several limitations in our study should be considered: (1) Real-world claims data can be subject to outliers, which can skew data on healthcare costs and lead to mean values that are higher than expected; (2) Services that are performed but not billed are not captured in this study; (3) Our data depend on professional *ICD-9/10* coding and the different coding patterns that are used in the clinical setting may not all be accurate – this is also true for other claims-based analyses [18]; and (4) Since this study did not assess treatment for MDD, the subcohorts likely contained a mix of appropriately treated and suboptimally treated/non-treated patients.

In addition, the results of this analysis may not be generalizable to non-commercial plans with potentially different populations and policies, such as Medicaid. Socioeconomic data that would support additional analyses are not available in the claims. Lastly, studies with large sample sizes may reveal small differences between groups that are determined to be statistically significant, but may not be clinically meaningful. As a result, small statistically significant differences that are observed in large studies should be evaluated for their clinical relevance.

## Conclusions

Patients with either T2D or CVD and comorbid MDD have higher total all-cause healthcare utilization and costs compared with similar patients without comorbid MDD. These findings reinforce the need for appropriate management of MDD in patients with T2D or CVD, which in turn may result in a decrease in healthcare utilization, as well as cost reductions for patients, payers, and the entire healthcare system. Further studies are warranted to elucidate whether higher healthcare resource utilization and costs in these patients are due to managing comorbid MDD itself or to managing more severe or less well-controlled T2D or CVD associated with comorbid MDD.

## Supplementary Information

**Additional file 1 Table S1.** Patient attrition. **Fig. S1.** Study design.

## Data Availability

The datasets generated during and/or analyzed during the current study are not publicly available since the research data contains protected patient health information and cannot be made available without violating privacy laws.

## Abbreviations

CVD: Cardiovascular disease
ED: Emergency department
HIPAA: Health Insurance Portability and Accountability Act
HMO: Health maintenance organization
HPA: Hypothalamic-pituitary-adrenal
*ICD-9/10-CM*: *International Classification of Diseases, Ninth or Tenth Revision, Clinical Modification*
MDD: Major depressive disorder
PHI: Protected Health Information
POS: Point of service
PPO: Preferred provider organization
SD: Standard deviation
T2D: Type 2 diabetes
US: United States
