# Supplementary material for: Incremental burden of comorbid major depressive disorder in patients with type 2 diabetes or cardiovascular disease: a retrospective claims analysis
Source: BMC Health Serv Res. 2021 Aug 6;21:778. doi: 10.1186/s12913-021-06802-9 (PMC8349073; doi:10.1186/s12913-021-06802-9)
Supplement: Supplementary file 1 — Additional file 1 Table S1. Patient attrition. Fig. S1. Study design. [file 12913_2021_6802_MOESM1_ESM.pdf]

Table S1 Patient Attrition

Measure	T2D		CVD	
	n	% Total members	n	% Total members
Total no. of patients in the study time frame: January 1, 2011, to September 30, 2018	12,800,000	100	12,800,000	100
A diagnosis of T2D or CVD on at least 2 separate claims within 12 months of each other	969,944	7.6	890,819	7.0
Commercially insured patients	146,282	1.1	136,632	1.1
Patients with T2D or CVD and MDD	11,753	0.1	14,750	0.1
Patients with T2D or CVD without MDD	134,529	1.1	121,882	1.0

CVD cardiovascular disease, MDD major depressive disorder, T2D type 2 diabetes.

Figure S1 Study Design

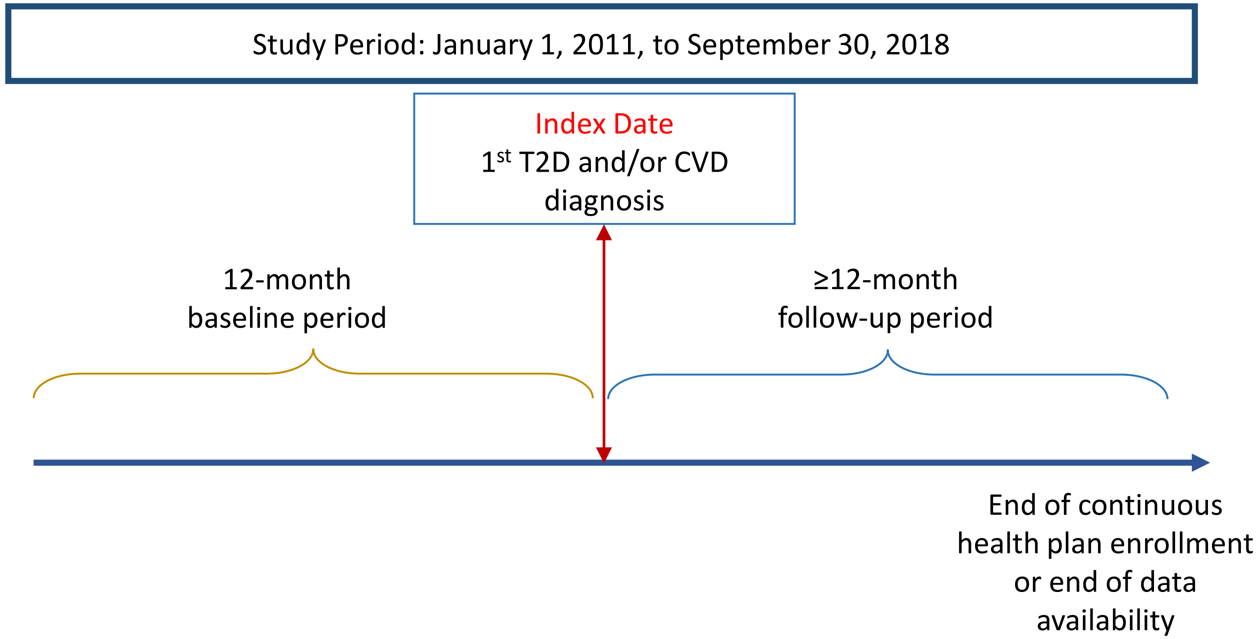

CVD cardiovascular disease, T2D type 2 diabetes.
